# Supplementary figures and images for: Glacial Legacies: Microbial Communities of Antarctic Refugia
Source: Biology (Basel). 2022 Oct 1;11(10):1440. doi: 10.3390/biology11101440 (PMC9598129; doi:10.3390/biology11101440)

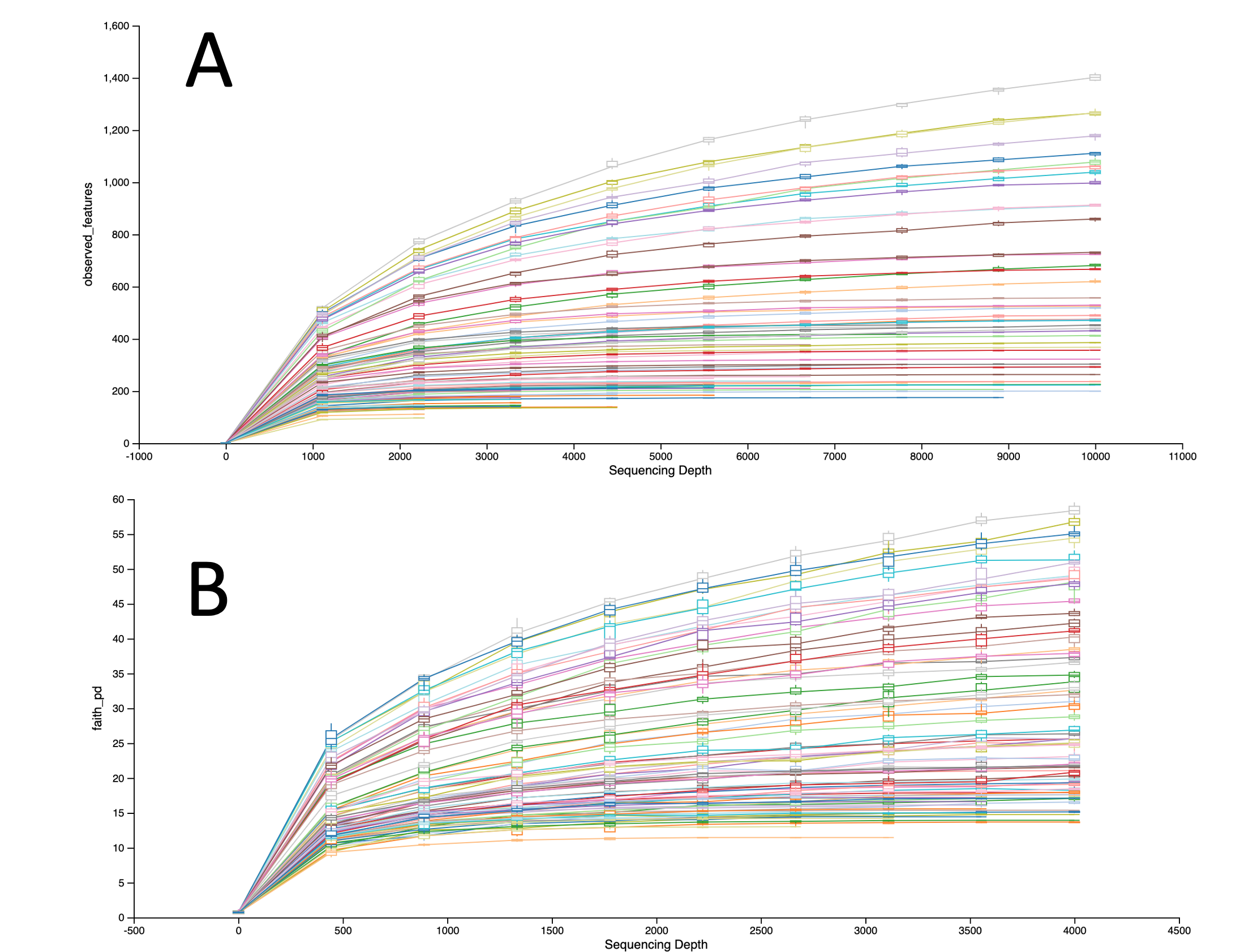

Supplement: Supplementary file 1 [file biology-11-01440-s001.zip › Sup Figure S1 - Alpha Rarefaction.png]

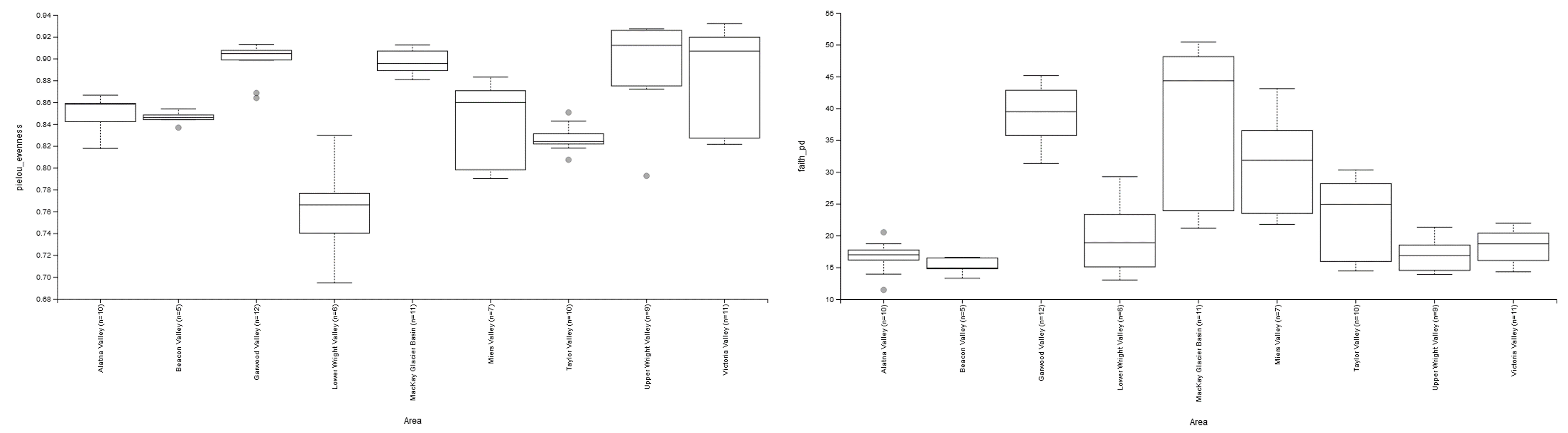

Supplement: Supplementary file 1 [file biology-11-01440-s001.zip › Sup Figure S2 - FaithPD and Evenness.png]

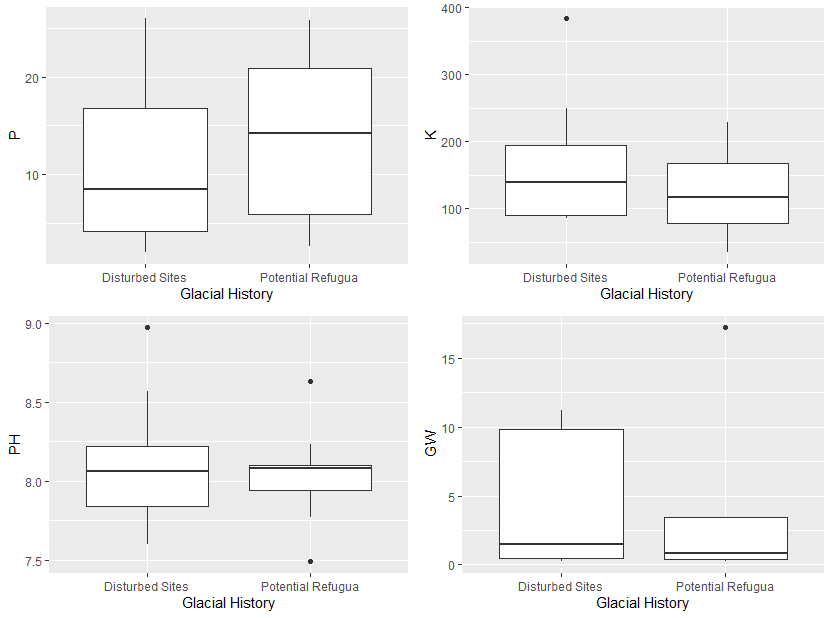

Supplement: Supplementary file 1 [file biology-11-01440-s001.zip › Sup Figure S3 - Environmental differences between glaciated sites and refugia sites.png]

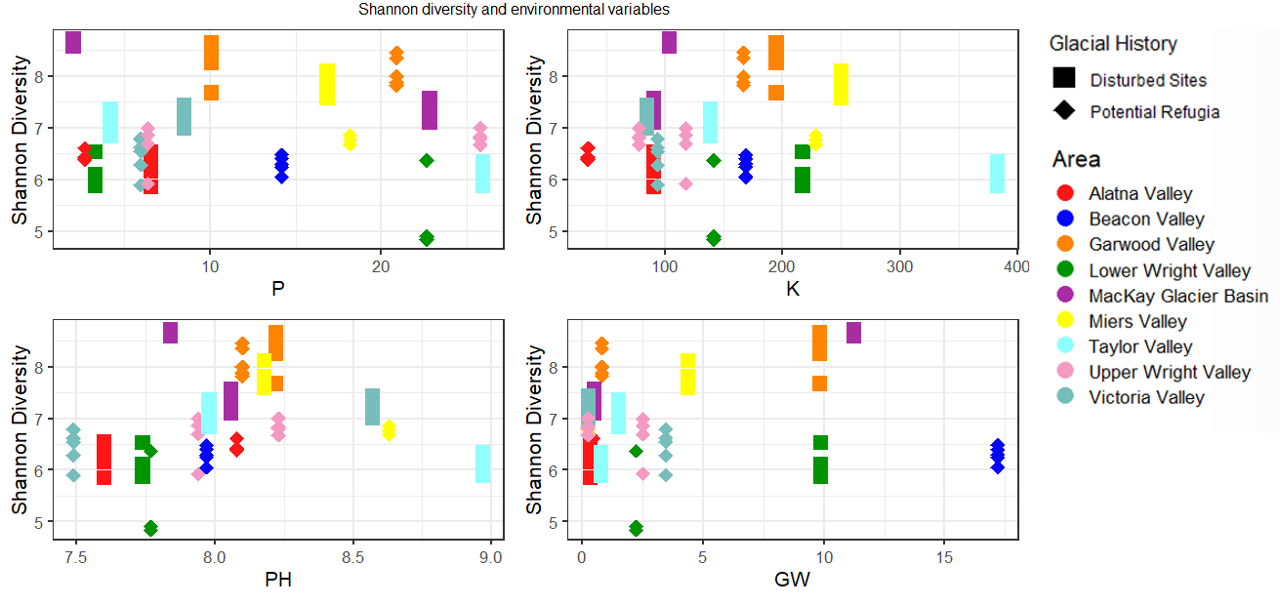

Supplement: Supplementary file 1 [file biology-11-01440-s001.zip › Sup Figure S4 - Other environmental variables.png]

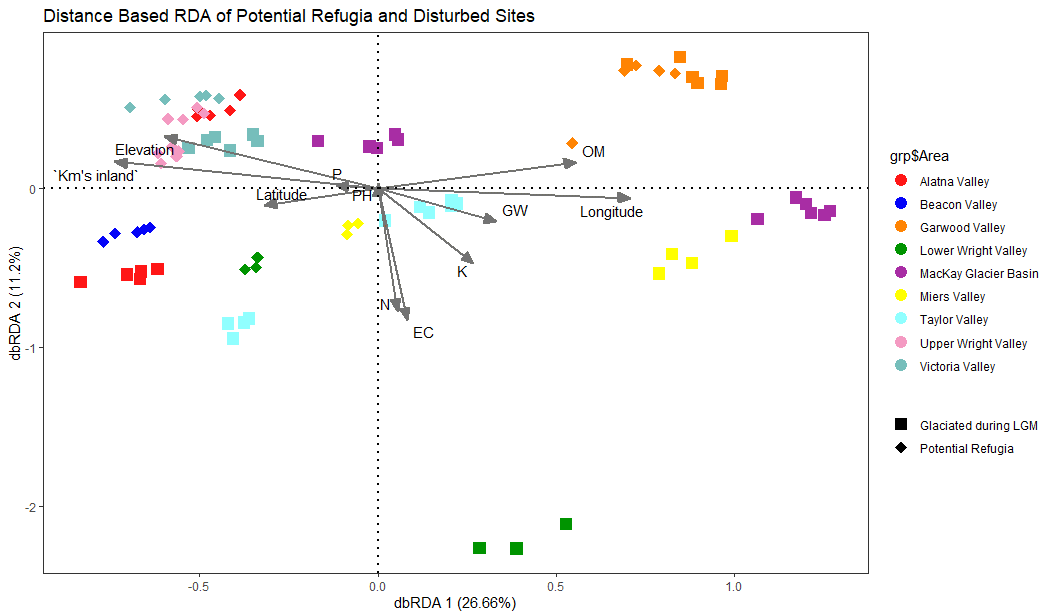

Supplement: Supplementary file 1 [file biology-11-01440-s001.zip › Sup Figure S5 - Distance-based Redundancy Analysis.png]

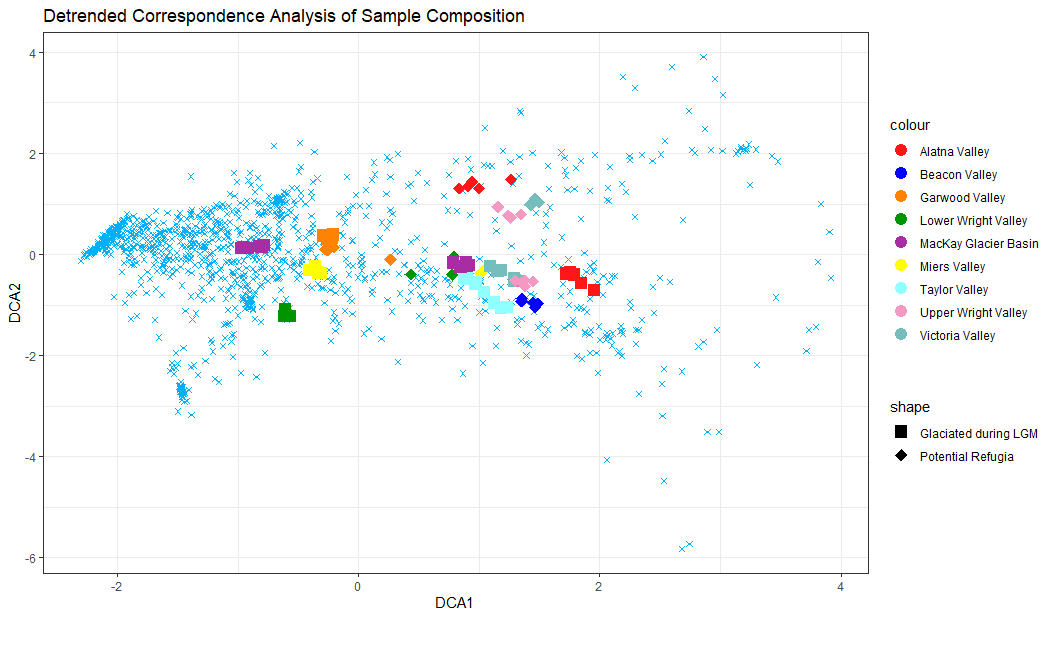

Supplement: Supplementary file 1 [file biology-11-01440-s001.zip › Sup Figure S6 - Detrended Correspondence Analysis.png]
